# Discoid Bicelles as Efficient Templates for Pillared Lamellar Periodic Mesoporous Silicas at pH 7 and Ultrafast Reaction Times

**DOI:** 10.1007/s11671-010-9813-9

**Published:** 2010-10-06

**Authors:** Paritosh Mohanty, Jinwoo Lee, Kerney Jebrell Glover, Kai Landskron

**Affiliations:** 1 Department of Chemistry, Lehigh University, 6 East Packer Avenue, Bethlehem, PA 18015, USA

**Keywords:** Bicelle, Mesoporous silica, Ultrasonication, Templating

## Abstract

We report the first synthesis of periodic mesoporous silicas templated by bicelles. The obtained materials form novel pillared lamellar structures with a high degree of periodic order, narrow pore size distributions, and exceptionally high surface areas.

## 

Hardly any other field of materials chemistry has experienced as much growth over the last 20 years as the field of periodic mesoporous materials [1]. Numerous applications have been found for periodic mesoporous materials in catalysis [2], separation [3], chemical storage [4], and delivery [5]. Periodic meosporous materials can be produced by either soft-templating [6] or hard-templating [4] methods. Soft-templating is the dominating technique because hard-templating requires additional undesirable reaction steps. With only one exception [7], all soft-templated periodic mesoporous materials are produced by templation of micelles. Most micellar templates are produced from cationic [6] or non-ionic [8] surfactants. Typical cationic surfactants are quaternized ammonium salts, for example cetyltrimethyl ammonium bromide (CTAB). Amphiphilic block-copolymers such as block-copolyethers have been extensively used as non-ionic surfactants. More recently, micelles from anionic surfactants have been used as templates in the presence of co-structure directing agents [9]. The use of new micellar templates has substantially driven the field of periodic mesoporous materials because new templates have frequently led to the discovery of new mesostructures with new pore systems [9]. With the exception of folic acid [7], no other templates but surfactant-containing micelles are known today for the production of periodic mesoporous materials. It has been attempted to use other soft templates, for example polyoxometallates [10], but to our best knowledge in no case periodic mesoporous materials have been obtained.

Bicelles form from two-component amphiphilic systems of one long-chain and one short-chain zwitter-ionic amphiphile namely zwitterionic dimyristoylphosphatidylcholine (DMPC) and dihexanoylphosphatidylcholine (DHPC) [11-13]. The distinguishing structural feature of a bicelle is a central planar bilayer formed by the long-chain phospholipid, surrounded by a rim of short-chain phospholipid that shields the long-chain lipid tails from water (Figure 1). The thickness of the discs is approximately two times the length of the DMPC molecule that is ca. 2 nm. The diameter of the discs can be adjusted to 5–40 nm by the variation in the molar ratio *q* of DMPC : DHPC [11]. Bicelles have been used so far primarily as hosts for membrane proteins. Because of their ability to align in magnetic fields, they have also been used extensively for NMR studies [12,14-19]. The features of bicelles open up intriguing new perspectives for templating, such as magnetic field–directed templating and immobilization of proteins in periodic mesostructures. The use of bicelles as templates for periodic mesoporous materials is furthermore appealing due to their unique discoid shape and the continuously adjustable diameter of the discoid from 5 to 40 nm. This is an enormous advantage over micelles that have a fixed diameter for a given surfactant. Therefore, the pore diameter of micelle-templated mesoporous silicas is fixed as well. Micelle expanders have been used for the preparation of periodic mesoporous silicas with increased pore sizes [8]. However, the pore size increase that can be achieved is relatively small. In principle, bicelles allow for a variation in pore diameter between 5 and 40 nm just by variation in the molar ratio of the two amphiphilic components. Except for one report about use of bicelles for the synthesis of platinum nanowheels, bicelles have not been employed as templates for materials synthesis at all [20]. Herein, we report for the first time the synthesis of a periodic mesoporous material from bicellar templates. This is only the second report that does not use a micellar template for the production of a periodic mesoporous material by soft-templating. The bicellar templates produce novel, pillared lamellar structures with exceptionally high surface areas. In contrast to micelles, which, with a few exceptions, require harsh acidic and basic conditions for the self-assembly, bicelles are very effective templates at pH 7. Bicelles are also the first zwitter-ionic templates ever used so far for the production of periodic mesoporous silicas.

**Figure 1:**
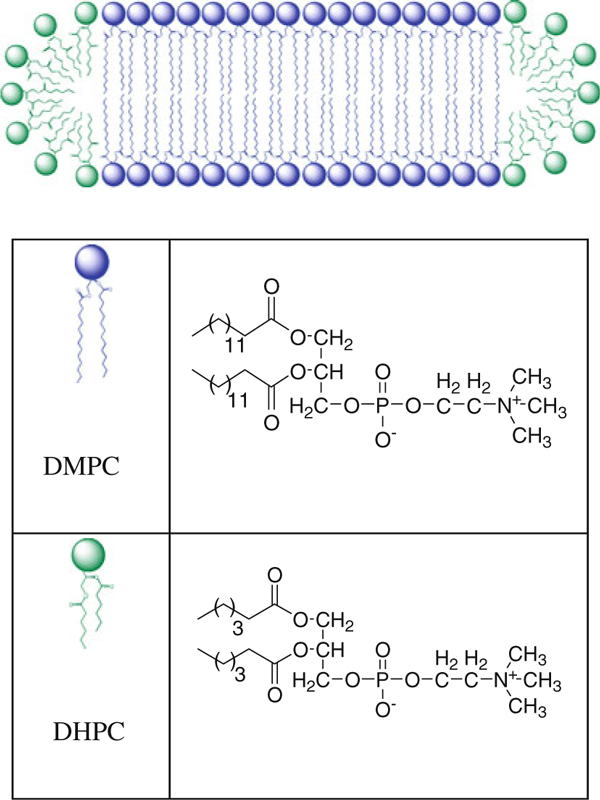
**Two-dimensional projection of bicelle and chemical structures of DMPC and DHPC**.

In the first experiment to produce bicelle-templated periodic mesoporous silicas, we added 218 mg of tetramethoxysilane (TMOS) to 2 ml of a 12% (w/w) aqueous solution of bicelles (*q* = 0.5) in the presence of 1 mg of NH_4_F as a catalyst. During the addition of the TMOS, the solution was constantly sonicated at room temperature. After the addition of the TMOS, a white precipitate formed immediately. The mixture was sonicated for another 2 min, and the precipitate was filtered off, washed with acetone, and vacuum-dried at room temperature. The dried sample (BMS-1) was investigated by small-angle powder X-ray diffraction (SAXS). The SAXS pattern (Figure 2a) shows a strong diffraction peak at *d* = 4.37 nm with a shoulder at 5.32 nm. Two higher-order peaks were observed at *d*-values of 3.09 and 2.52 nm. In order to remove the template to produce a periodic mesoporous material, we calcined the material at 500°C. However, we observed a complete loss of mesostructure according to SAXS. To remove the template at milder conditions, we extracted BMS-1 with a methanol/chloroform mixture, which is known as a good solvent for both DMPC and DHPC. However, also extraction at room temperature led to complete loss of mesostructure (Figure 2b). This suggests that a lamellar structure is present.

**Figure 2:**
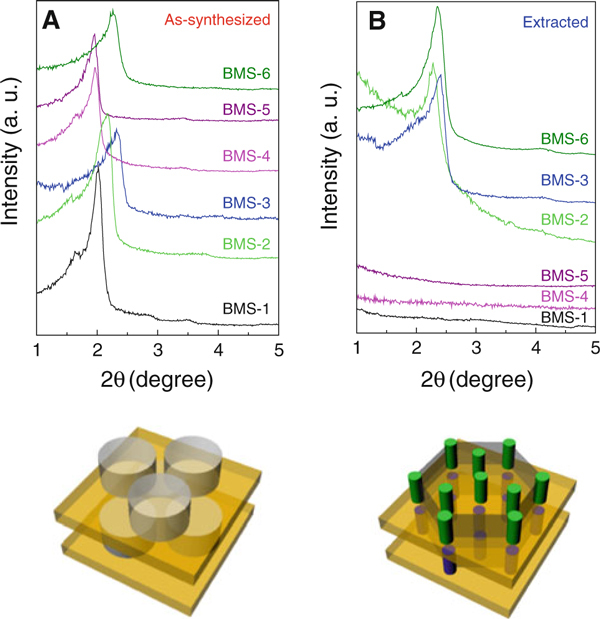
**Small angle X-ray diffraction patterns (*top*) and cartoons of bicelle-templated silica structures (*bottom*)**. *Bottom left*: As-synthesized BMS-1 structure. *Bottom right*: Extracted BMS-2 structure. *Grey*: bicelles, Orange: silica layers, *Green* and *blue*: silica pillars.

The formation of a lamellar structure from bicelles as templates can be easily understood considering the discoid shape of the bicelles. The discs close-pack hexagonally to form layers. Each layer of bicelles is sandwiched between two silica layers. This structure is similar to a hexagonal pillared lamellar structure (HPL) recently reported by Stucky et al. for a periodic mesoporous organosilica [21]. In comparison with the material reported by Stucky et. al., the hexagonally packed silica pillars are replaced by hexagonally packed bicelles [21]. The structural similarity between the HPL material and BMS-1 can be confirmed by comparison of the SAXS patterns. The reflexes of BMS-1 at 5.32, 4.37, 3.09, and 2.52 nm can be indexed as (100), (002), (110), and (103) lattice planes, respectively. The (110) and (103) higher-order peaks were not well resolved, which indicates the presence of stacking faults. These lattice planes are consistent with a hexagonal lamellar structure of P6_3_/mmc symmetry and lattice parameters, *a* = 6.14 and *c* = 8.74 nm. Table 1 lists the structural parameters of BMS materials (extracted).

**Table 1:** Structural parameters of BMS materials (extracted)

Sample	Lattice constants (nm)		**BET surface area (m**^**2**^**/g)**	**Pore size (nm)**^**a**^	**Pore volume (cm**^**3**^**/g)**
				
	*a*	*c*			
BMS-2	5.53	7.74	1,074	2.5	0.72
BMS-3	5.02	7.35	1,701	2.7	1.28
BMS-6	5.82	7.51	1,191	2.4	0.82

^a^ Pore size distribution was calculated from the desorption branch using the BJH method

Layers of hexagonally packed bicelles contain holes between the bicelles. In the following, we increased the TMOS/bicelle ratio to make the formation of silica pillars in the holes between the bicelles more likely. To do so, we performed an additional experiment in which the bicelle amount was decreased by decreasing the volume of the 12% (w/w) bicelle solution to 1.0 ml. The same amount of TMOS (218 mg) was added, thereby doubling the TMOS concentration in the bicelle solution. SAXS showed a periodic mesostructure for the product material similar to BMS-1 (Figure 2a). The reflections have *d*-spacings of 5.62 (001), 4.06 (002), and 2.37 (103), suggesting again a hexagonal lamellar structure. The lattice constants are *a* = 6.49 nm and *c* = 8.13 nm. The material (BMS-2) could be extracted with methanol/chloroform under the preservation of mesoscopic order. SAXS of the extracted sample showed a single, relatively broad reflex at *d* = 3.87 nm with a shoulder at 4.62 nm, indicating that this material has lost some order during the template removal process. The lattice constant of the extracted material is *a* = 5.53 and *c* = 7.74 nm, indicating that the mesostructure has shrunk during the extraction process. The SAXS data suggest that silica pillars have grown though the holes between the bicelles, and a pillared lamellar structure has formed. This pillared lamellar structure is different from the HPL material reported by Stucky et. al. In contrast to the HPL material, the silica pillars are not hexagonally close-packed but form arrays of edge-sharing hexagons. Thus, BMS-2 is a new periodic mesoporous silica structure type. N_2_ sorption of BMS-2 shows a type IV isotherm (Figure 3a).

**Figure 3:**
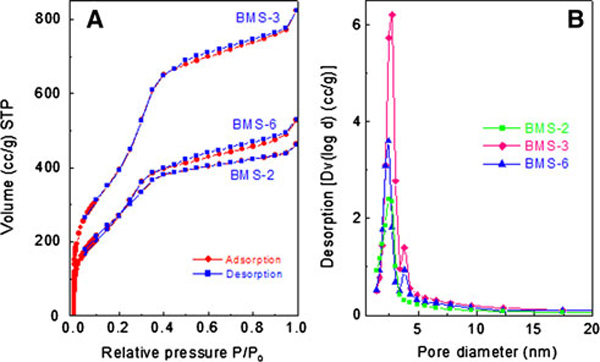
**a N_2_ sorption isotherms, and b PSD of BMS samples**. The PSD were calculated from the desorption branch of the isotherm using BJH method.

The capillary condensation step was observed at pressures between ca. 0.2 and 0.35 p/p_0_. The pore size distribution calculated from the desorption isotherm revealed the pore size of ca. 2.5 nm. This pore diameter reflects roughly the thickness of the bicelles. The fact that the pore diameters are somewhat smaller than the thickness of the bicelles may be explained by (a) penetration of the hydrophilic part of the bicelle by the silica framework, (b) interpenetration of hydrophobic chains in the bicelle core, and (c) pore contraction upon template removal. These effects are also often observed in micelle-templated silicas. For example, cetyltrimethylammonium bromide (CTAB) has a chain length of 2.2 nm. However, mesoporous silicas produced by CTAB micelles have typical diameters of only 2.5–3.0 nm [8]. The fact that the lattice parameter a (a = 5.33 nm) of BMS-2 is smaller than the bicelle diameter (ca. 8 nm) can be explained by similar effects. The BET surface area of the material is 1,074 m^2^ g^-1^. Transmission electron microscopy (TEM) data confirm the presence of a mesostructure (Figure 4a); however, no clear periodicity could be observed, which is consistent with the relatively broad SAXS reflexes.

**Figure 4:**
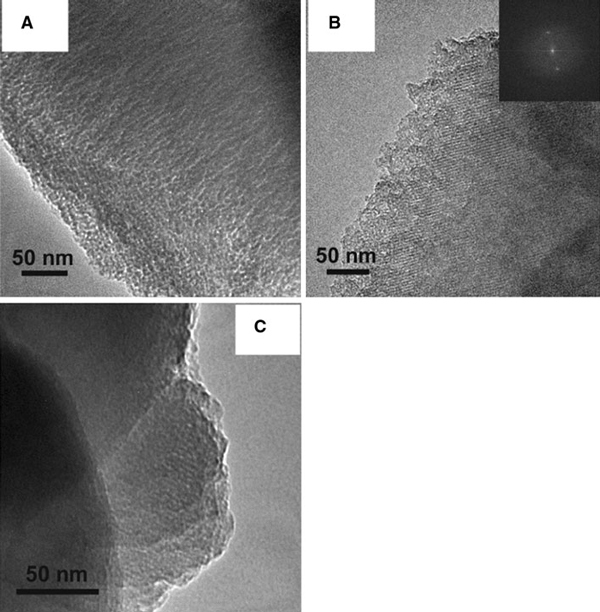
**TEM images of a BMS-2, b BMS-3, and c BMS-6**. The inset of figure **b** shows the corresponding Fourier transform images.

To understand the relationships between the reaction conditions and the structural properties of the BMS materials in more depth, we performed two additional experiments with bicelle concentrations of 5% (w/w) and 20% (w/w), respectively. The TMOS/bicelle ratio was kept constant by variation in the volume of the bicelle solution (see additional file 1 for details). For both as-synthesized materials (BMS-3 and BMS-4), strong reflexes at *d* = 3.80 and 4.50 nm, with two shoulders at 4.01 and 5.38, respectively, were observed by SAXS (Figure 2a). One higher-order peak was observed at 2.59 nm for BMS-4. However, no clear higher-order peak was observed for the sample BMS-3.

While BMS-3 retained mesoscopic periodicity upon extraction (*d* = 4.348, 3.75, 2.12, and 1.85 nm, *a* = 5.02, *c* = 7.35), the mesostructure was lost for BMS-4 (Figure 2b). This suggests that BMS-4 is isotypic to BMS-1. N_2_ sorption of BMS-3 produced a type IV isotherm, which confirms the presence of a mesostructure in the material (Figure 3a). Similar to BMS-2, the capillary condensation takes place at pressures between 0.20 and 0.35 p/p_0_. The BJH pore size distribution calculated from the desorption branch of the isotherm is narrow and centred around 2.7 nm (Figure 3b). This pore size is very similar to the pore size of BMS-2 and suggests a pillared lamellar structure, which is isotypic to BMS-2. The pore volume of BMS-3 was found to be very high (1.28 cm^3^ g^-1^). The BET surface area of BMS-3 is exceptionally high for a periodic mesoporous silica material (1,701 m^2^ g^-1^). The much higher surface area of BMS-3 in comparison with BMS-2 can be explained by the decreased thickness of the silica layers. According to SAXS and sorption data, the thickness of the silica layers are estimated to be only 1.0 nm compared with 1.4 nm (BMS-2). TEM and FFT of BMS-3 confirm the mesostructural order of BMS-3 (Figure 4b). The lattice spacings of the TEM images (3.7 nm) are half of the lattice parameter *c* and thus in excellent accordance with SAXS data.

In a further series of experiments, we changed the TMOS/bicelle ratio by variation in the bicelle concentration in the aqueous solution. Compared to the synthesis of BMS-1 (12% (w/w) bicelles), 20 and 5% (w/w) bicellar solutions were used, yielding the materials BMS-5 and BMS-6, respectively. In both cases, well-ordered mesostructures were observed by SAXS, showing *d*-spacings at 4.80 nm (001), 4.50 nm (002), and 2.59 (103) for BMS-5 and 5.04, 3.91, and 2.25 nm for BMS-6 nm, respectively (Figure 2a). As expected, the mesostructural order vanished after extraction of BMS-5 due to the very low TMOS/bicelle ratio (same ratio as BMS-1). For BMS-6, with a higher TMOS/bicelle ratio, the mesostructural periodicity was preserved (Figure 2b), and the lattice constants of could be determined (*a* = 5.82 nm, *c* = 7.51 nm). A well-defined type IV N_2_ isotherm was obtained that was similar to BMS-2 and BMS-3 (Figure 3a). The BJH pore size distribution of BMS-6 was centred around 2.4 nm, which is similar to BMS-2 and BMS-3. The pore volume of BMS-6 (0.82 cm^-3^ g^-1^) is similar to BMS-3. The BET surface area of BMS-6 is 1,191 m^2^ g^-1^. TEM clearly confirms the presence of a periodic mesostructure, showing lattice fringes with spacings of ca. 3.8 nm which is in excellent accordance with SAXS data (Figure 4c).

In summary, bicelles are very efficient templates for periodic mesoporous silicas. The synthesis can be done in pure water (containing catalytic amounts of NH_4_F) at room temperature within a reaction time of 2 min. The material structures derive from lamellar structures. For BMS-1, BMS-4, and BMS-5, non-pillared hexagonal lamellar structures are formed, while BMS-2, BMS-3, and BMS-6 form a novel hexagonal pillared lamellar structure. The material BMS-3 shows a very high surface area of 1,701 m^2^ g.

## Supplementary Material

Additional file 1 Click here for file
