# Supplementary material for: Discoid Bicelles as Efficient Templates for Pillared Lamellar Periodic Mesoporous Silicas at pH 7 and Ultrafast Reaction Times
Source: Nanoscale Res Lett. 2010 Oct 6;6(1):61. doi: 10.1007/s11671-010-9813-9 (PMC3212208; doi:10.1007/s11671-010-9813-9)
Supplement: Additional file 1 [file 1556-276X-6-61-S1.docx]

SUPPORTING INFORMATION

**Experimental section:**

***Chemicals*:**

Tetramethoxysilane (98 %) Alfa Aesar, Ammonium Fluoride (98 %) Alfa Aesar. All the chemicals were used as-received without further purification. 1,2-Dimyristoyl-*sn*-Glycero-3-Phosphocholine (DMPC) and 1,2-Dihexanoyl-*sn*-Glycero-3-Phosphocholine (DHPC) were obtained as powder by Aanti Polar Lipids. (Alabaster, AL)

***Synthesis of Bicelle solution***

Synthesis of Bicelle solution

| Material | ***q*** | %(w/w) | Total (mL) | X (mg) of DMPC | Y(µL) of Water | Z(µL) of DHPC |
| --- | --- | --- | --- | --- | --- | --- |
| BMS-1 | 0.5 | 12 | 2.0 | 102.66 | 1348 | 532 |
| BMS-2 | 0.5 | 12 | 1.0 | 51.33 | 647 | 266 |
| BMS-3 | 0.5 | 5 | 3.6 | 76.99 | 3111 | 399 |
| BMS-4 | 0.5 | 20 | 0.9 | 76.99 | 411 | 399 |
| BMS-5 | 0.5 | 20 | 1.5 | 128.32 | 685 | 665 |
| BMs-6 | 0.5 | 5 | 1.5 | 32.08 | 1296 | 166 |

X mg of DMPC was resuspended with Y ml of HPLC grade water and voltexed until homogeneous milk-like suspension is obtained. Z ml of 25% (w/w) DHPC were slowly added into resuspended DMPC while mixing. After an extra one minute of voltexing, clear bicelle solution is obtained. The solution was spun for one hour at 20,000xg to remove bubbles from the solution.

***Synthesis of BMS materials:***

In a typical synthesis procedure, about 1 mg of ammonium fluoride was dissolved in *x* ml of bicelle solution with a concentration of *y* % (w/w). To it *x* ml 218 mg of the tetramethoxysilane (TMOS) was added. During the addition of the tetramethoxysilane, the bicelle solution was continuously sonicated at room temperature. After the addition of the TMOS a white precipitate formed immediately. The mixture was sonicated for another 2 min and the precipitate was filtered off, washed with acetone, and vacuum-dried at room temperature.

| **Material** | ***x* (ml)** | ***y* (%)** |
| --- | --- | --- |
| BMS-1 | 2 | 12 |
| BMS-2 | 1 | 12 |
| BMS-3 | 3.6 | 5 |
| BMS-4 | 0.9 | 20 |
| BMS-5 | 1.5 | 20 |
| BMS-6 | 1.5 | 5 |

**Characterization of the Materials:**

The formation of the ordered mesostructure, study of its structure and microstructures were carried out by small angle x-ray diffraction (SAXS) and transmission electron microscopy (TEM). The mesoporous structure of the BMS samples were studied by nitrogen sorption analysis. SAXS patterns of the specimens were obtained using a Rigaku Rotaflex diffractometer with a Cu Kα radiation source (λ = 0.15405 nm). The TEM images were taken on a JEOL JEM-2000 electron microscope operated at 200 kV. Sample for the TEM analysis was prepared by dispersing the particles in acetone and dropping a small volume of it onto a holey carbon film on a copper grid. The N_2_ adsorption/desorption isotherm was measured at 77 K using an Autosorb-1 instrument (Quantachrome). Prior to the measurement, the specimens were out-gassed at 120 ºC for 2 h.
